# The association between bone mineral density and postoperative drainage volume following cruciate-substituting primary total knee arthroplasty: a cross-sectional study

**DOI:** 10.1186/s43019-021-00107-1

**Published:** 2021-07-28

**Authors:** Yuthasak Peerakul, Jirapong Leeyaphan, Karn Rojjananukulpong

**Affiliations:** Bamrasnaradura Infectious Diseases Institute, Department of Disease Control, Ministry of Public health, 38 Talat Khwan, Mueang, Nonthaburi, 11000 Thailand

**Keywords:** Total knee arthroplasty, Bone mineral density, Blood loss, Factor

## Abstract

**Background:**

The prevalence of osteoporosis in patients who undergo a primary total knee arthroplasty (TKA) is increasing. Low bone mineral density (BMD) is related to unfavorable outcomes following TKA such as migration of uncemented tibial components. Postoperative blood loss in TKA is an important complication. Non-modifying predicting factors for postoperative blood loss in patients undergoing primary TKA need further elucidation. Studies on the association between BMD and blood loss after TKA are limited. We aimed to demonstrate the relationship between BMD and postoperative drainage volume following primary TKA.

**Methods:**

A cross-sectional study was conducted between January 2014 and August 2020. A total of 119 primary varus osteoarthritis knees with BMD results were included in the study. Patients with secondary causes of osteoporosis were excluded.

**Results:**

The median postoperative drainage volume of participants in the normal total hip BMD group and the normal trochanter BMD group was higher than that of patients in the low total hip BMD group and the low trochanter BMD group (285.0 ml vs 230.0 ml, *P* = 0.003; 282.5 ml vs 240.0 ml, *P* = 0.013, respectively). Multivariate regression analyses showed that operative time, spinal anesthesia, and normal total hip BMD status were significant predictive factors associated with increased postoperative drainage volume (*P* = 0.014, 0.022, and 0.013, respectively). No association was identified between the lumbar spine BMD status and postoperative drainage volume.

**Conclusions:**

The relationship between BMD and postoperative blood loss in primary TKA was identified in this study. Normal total hip BMD was found to be associated with an increased postoperative drainage volume after primary TKA compared with low BMD.

## Background

Blood loss following total knee arthroplasty (TKA) is a common concerning postoperative complication. The type of anesthesia used [1], type of surgery, operative time, body mass index (BMI) < 27 kg/m^2^ [2], prosthesis design, and postoperative anticoagulation drugs given [3] were found to be predictive of postoperative blood loss after TKA. Despite the potential for some influencing factors to be reduced in the preoperative period, there are still many predisposing factors that cannot be preoperatively modified, such as older age, sex, hypertension, the presence of rheumatoid arthritis, and an American Society of Anesthesiologists (ASA) score > 3 [2]. Thus, many medication and methods have been investigated to reduce postoperative blood loss and blood transfusions, including tranexamic acid [4–7], computer-assisted surgery [8–10], closed suction drainage [11, 12], no drainage [13], fibrin sealants [14], intramedullary femoral canal sealing [15], compression dressings [12], and a postoperative high-flexion knee position [16].

Osteoporosis is increasingly being found in patients who have undergone a primary TKA [17]. A previous study reported a prevalence of osteoporosis of 50.0% in patients awaiting TKA [18]. A low bone mineral density (BMD) was reported to be associated with unfavorable outcomes following TKA, such as the migration of uncemented tibial components [19]. The association between BMD and the volume of blood loss is controversial. A recent study showed an association between BMD and perioperative blood loss during mini-invasive posterior spinal fusion surgery. The patients who had a low lumbar spine BMD tended to have more perioperative blood loss than those with a normal BMD [20]. However, one study demonstrated no difference in the postoperative drainage volume after TKA in patients with rheumatoid arthritis, which is an osteoporosis risk factor [21], compared with normal cases with primary osteoarthritis [22]. An extensive literature review identified no previous reports on the relationship between BMD and postoperative blood loss in patients with primary TKA. This study aimed to clarify the association between BMD and the postoperative drainage volume following primary TKA. Risk factors for postoperative drainage volume were also identified and analyzed.

## Methods

### Study population

This cross-sectional study enrolled participants diagnosed with varus knee osteoarthritis who underwent a primary TKA with a single surgeon (YP) from January 2014 to August 2020. Participants who were diagnosed with chronic liver disease or chronic kidney disease were excluded. In addition, participants who lacked BMD results were also excluded. The study protocol was approved by the Institutional Review Board (S059h/63_ExPD).

### Data collection

Data on age, sex, underlying hypertension, BMI, anesthesia, ASA score, preoperative hemoglobin content, preoperative platelet count, operative time, 24-h postoperative mean arterial pressure (MAP), 24-h postoperative blood loss drainage volume [23], BMD, and blood transfusions were collected from medical records and included in the analysis.

Dual-energy X-ray absorptiometry (DXA) was performed at two sites: the lumbar spine (L1–L4) and the hip (femoral neck, trochanter, and total hip). All participants were assessed using the same DXA machine (Discovery Wi, Hologic, Marlborough, MA, USA). The T-score interpretation used Asian matched values. The DXA results were interpreted according to the World Health Organization criteria. T-scores of ≤ − 2.5 standard deviations (SD) below the reference mean were categorized as indicative of osteoporosis, T-scores between − 1.0 and − 2.5 SD as osteopenia, and T-scores ≥ − 1.0 SD as normal. In this study, patients with T-scores <− 1.0 SD were categorized as having a low BMD, while those with T-scores ≥ − 1.0 SD were labeled as having a normal BMD.

### Surgical technique and postoperative management

All TKAs were performed by a single surgeon to reduce the surgical technique bias. Patients were instructed to stop antiplatelet drugs 7 days before surgery. A tourniquet with a pressure of 300 mmHg was used in all cases. A full-time tourniquet was used in all TKAs. The surgeries were performed after positioning. The lower extremity was prepared and draped using a standard method. The limb was elevated and exsanguinated with an elastic bandage. A straight midline skin incision was made, starting from 5 cm proximal to the superior pole of the patella and continuing to the medial side of the tibial tuberosity. A medial parapatellar arthrotomy was made, and then the medial soft tissue was released subperiosteally from the proximal medial part of the tibia to the posteromedial corner of the tibia. The patella was then everted after releasing the patellofemoral ligament. The anterior and posterior cruciate ligaments were released. The medial and lateral menisci were excised, and then the proximal portion of the tibia was resected at 90 ° to its long axis with an intramedullary tibial guide. The distal femur was resected at 5 ° valgus with an intramedullary femoral guide. The selective patella was then prepared for resurfacing. Anterior, posterior, chamfer, and box cuts were made. The trial components were placed to test the knee for stability and for adequate patellar tracking. The knee was irrigated with 1000 ml of normal saline, and the bone was dried. There was no femoral bone plug to close femoral guide hole. The components (New Wave™, Groupe Lépine, Genay, France) were cemented into place. Two grams of tranexamic acid was administered directly into the joint space. The drain was placed, and then the quadriceps tendon and joint capsule were repaired with an interrupted absorbable suture (number 1). There was no period of releasing the tourniquet for bleeding control. The skin was closed with absorbable subcuticular running sutures (number 3/0). The knee was dressed in gauze without a bandage. The tourniquet was deflated, and then the drain was released. All patients received patient-controlled analgesia for 48 h without postoperative deep vein thrombosis prophylaxis. Because of the low prevalence of fatal pulmonary embolism after TKA [24] and the risk of bleeding, postoperative thromboprophylaxis was not used in this study. The drain was removed at 24–48 h post-operation.

### Statistical analysis

A sample size calculation was performed by using G*Power 3.1 software (University of Dusseldorf, Dusseldorf, Germany) [25], which determined that a minimum of 118 participants was required with an effect size of 0.15 to achieve a statistical power ≥ 80 at *P*-value α levels of ≤ 0.05 and for 10 predictors. Descriptive analyses such as mean values with SDs, medians with the 25^th^–75^th^ percentile ranges, or counts were performed. Independent sample *t* tests were used to compare means of normally distributed data, and the Mann−Whitney *U* test was used for non-normally distributed continuous variables. Pearson’s correlation was used in the analyses. A univariate regression analysis was performed to estimate the relationships among variables and the postoperative drainage volume. In terms of qualitative variables, for sex, male was set as “1” and female was set as “0”. For hypertension, yes was set as “1” and no was set as “0”. For anesthesia, spinal anesthesia was set as “1” and general anesthesia was set as “0”. For BMD status, low was set as “1” and normal was set as “0”. The variables with *P* values of less than 0.25 in the univariate regression analysis or other variables of known clinical relevance were included for further multivariate regression analysis [26]. All data were analyzed using the Statistical Package for the Social Sciences for Windows 26.0 (Armonk, NY, USA). A *P* value of < 0.05 was considered statistically significant.

## Results

**Fig. 1:**
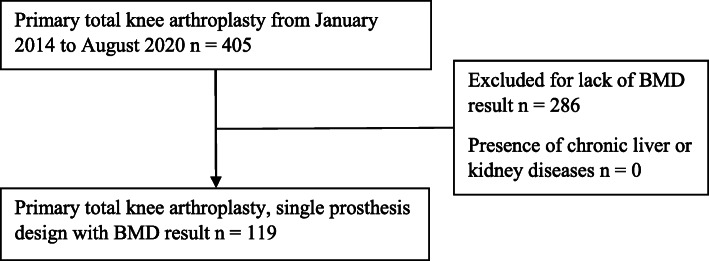
Flowchart for subject enrollment

**Fig. 2:**
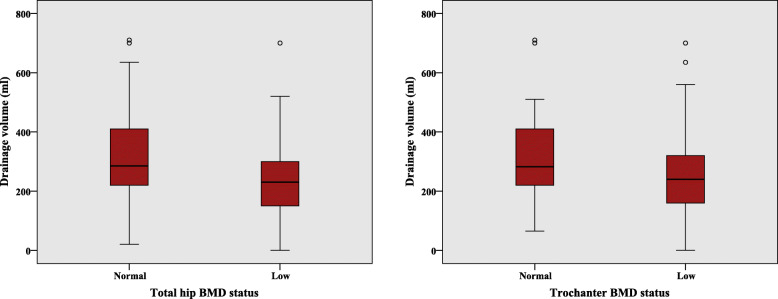
Box plots of 24-h postoperative drainage volume and BMD status. Outliers are indicated by circles

Initially, 405 participants were to be included in the study, but 286 were excluded because of a lack of BMD results. The remaining 119 participants were included in the present study (Fig. 1). The clinical characteristics of the study population are shown in Table 1. The median postoperative drainage volume of participants in the normal total hip BMD group and the normal trochanter BMD group was higher than that in the low total hip BMD group and the low trochanter BMD group (*P* = 0.003, *P* = 0.013, respectively) (Table 2, Fig. 2). The total hip T-score was analyzed and was found to have significant correlation with the postoperative drainage volume (*P* = 0.05).

**Table 1:** Clinical characteristics of the study population categorized by total hip T-score

Clinical characteristics	Normal BMD (***n*** = 69)	Low BMD (***n*** = 50)	***P*** value
Age (years)	72.64 ± 5.95	73.90 ± 5.67	0.247
Sex			
Female	62 (89.9%)	48 (96.0%)	0.211
Male	7 (10.1%)	2 (4.0%)	
BMI (kg/m^2^)	27.77 ± 4.19	25.50 ± 3.64	0.002
Anesthesia			
Spinal anesthesia	20 (29.0%)	16 (32.0%)	0.724
General anesthesia	49 (71.0%)	34 (68.0%)	
ASA score			
2	28 (40.6%)	14 (28.0%)	0.156
3	41 (59.4%)	36 (72.0%)	
Hypertension			
Yes	54 (78.3%)	43 (86.0%)	0.283
No	15 (21.7%)	7 (14.0%)	
Prior antiplatelet treatment			
Yes	8 (11.6%)	7 (14.0%)	0.696
No	61 (88.4%)	43 (86.0%)	
Operative time (min)	103.67 ± 8.29	96.70 ± 8.93	< 0.001
Patella resurfacing			
Yes	59 (85.5%)	36 (72.0%)	0.07
No	10 (14.5%)	14 (28.0%)	
Blood transfusion			
Yes	2 (2.9%)	1 (2.0%)	0.758
No	67 (97.1%)	49 (98.0%)	
Postoperative MAP (mmHg)	96.70 ± 9.40	95.40 ± 10.71	0.485
Laboratory parameters			
Hemoglobin (g/mm^3^)	12.1 ± 1.2	12.2 ± 1.2	0.787
Hematocrit (%)	37.3 ± 3.7	37.4 ± 3.3	0.884
Platelet (× 10^3^/mm^3^)	261.2 ± 70.3	246.4 ± 52.0	0.213

Variables are presented as mean ± SD or number (count)

*BMD* bone mineral density, *BMI* body mass index, *ASA* American Society of Anesthesiologists, *MAP* mean arterial pressure

**Table 2:** Comparison between postoperative drainage volume (ml) with BMD site and bone status

Site	Normal BMD	Low BMD	***P*** value
Lumbar spine	(*n* = 56) 245.0 (172.5, 330.0)	(*n* = 57) 270.0 (190.0, 350.0)	0.836
Trochanter	(*n* = 58) 282.5 (217.5, 410.0)	(*n* = 61) 240.0 (160.0, 320.0)	0.013
Femoral neck	(*n* = 22) 260.0 (217.5, 376.3)	(*n* = 97) 270.0 (175.0, 340.0)	0.549
Total hip	(*n* = 69) 285.0 (215.0, 410.0)	(*n* = 50) 230.0 (150.0, 305.0)	0.003

Variables are presented as medians (the 25^th^ percentile, the 75^th^ percentile)

*BMD* bone mineral density

The univariate regression analysis showed significant associations of the operative time, trochanter BMD status, and total hip BMD status with the postoperative drainage volume (all *P* < 0.05) (Table 3). No relationships were identified between the postoperative drainage volume and femoral neck BMD status or lumbar spine BMD status.

**Table 3:** Univariate regression analysis of the relationships between variables and the postoperative drainage volume

Variables	β (SE)	***t***	***P***	***R***^**2**^
Age (years)	0.009 (0.213)	0.100	0.920	0.000
Sex	0.061 (48.631)	0.663	0.508	0.004
BMI (kg/m^2^)	0.054 (3.142)	0.581	0.562	0.003
Hypertension	− 0.012 (33.179)	−0.224	0.823	0.000
Anesthesia (SA vs GA)	0.100 (27.905)	1.087	0.279	0.010
Operative time (min)	0.305 (1.339)	3.465	0.001	0.093
Postoperative MAP (mmHg)	−0.102 (1.294)	−1.109	0.270	0.010
Hemoglobin (g/mm^3^)	−0.047 (10.497)	−0.505	0.614	0.002
Platelet (×10^3^/mm^3^)	0.028 (0.204)	0.299	0.766	0.001
Lumbar spine status (normal vs low)	−0.027 (25.913)	−0.287	0.775	0.001
Femoral neck status (normal vs low)	−0.032 (33.168)	−0.350	0.727	0.001
Trochanter status (normal vs low)	−0.219 (25.150)	−2.423	0.017	0.048
Total hip status (normal vs low)	−0.277 (25.075)	−3.124	0.002	0.077

*SA* spinal anesthesia, *GA* general anesthesia

A multivariate regression analysis was used to correct for operative time, trochanter BMD status, and total hip BMD status, which were identified as significant independent factors in the univariate regression analysis. Age, sex, BMI, hypertension, and anesthesia were analyzed in the model because of their clinical relevance to postoperative blood loss. Femoral neck BMD status, trochanter BMD status, and total hip BMD status were analyzed in a separate model because of multicollinearity problems. The multivariate analysis using total hip BMD status showed that spinal anesthesia, the operative time, and the total hip BMD status were significantly associated with the postoperative drainage volume (*P* = 0.022, 0.014, and 0.013, respectively). However, the multivariate regression analysis using the femoral neck BMD status and trochanter BMD status model found that neither femoral neck BMD status nor trochanter BMD status were related to the postoperative drainage volume (*P* = 0.853 and *P* = 0.109, respectively) (Table 4).

**Table 4:** Multivariate regression analysis of the relationships between variables and the postoperative drainage volume

Variables	Β (SE)	***t***	***P***	VIF
Total hip status (*R*^2^ = 0.449; adjusted *R*^2^ = 0.202)				
Anesthesia (SA vs GA)	0.218 (28.317)	2.328	0.022	1.105
Operative time (min)	0.269 (1.582)	2.514	0.014	1.447
Lumbar spine status (normal vs low)	0.103 (27.191)	1.030	0.306	1.254
Total hip status (normal vs low)	−0.272 (29.815)	−2.530	0.013	1.465
Trochanter status (*R*^2^ = 0.415; adjusted *R*^2^ = 0.173)				
Anesthesia (SA vs GA)	0.211 (28.960)	2.210	0.029	1.115
Operative time (min)	0.311 (1.580)	2.914	0.004	1.392
Lumbar spine status (normal vs low)	0.052 (26.722)	0.534	0.595	1.169
Trochanter status (normal vs low)	−0.170 (28.697)	−1.617	0.109	1.348
Femoral neck status (*R*^2^ = 0.389; adjusted *R*^2^ = 0.151)				
Anesthesia (SA vs GA)	0.227 (29.177)	2.354	0.020	1.103
Operative time (min)	0.362 (1.561)	3.432	0.001	1.325
Lumbar spine status (normal vs low)	0.017 (27.473)	0.165	0.869	1.204
Femoral neck status (normal vs low)	0.020 (37.517)	0.186	0.853	1.360

All models were adjusted for age, sex, body mass index, hypertension, postoperative mean arterial pressure, hemoglobin, and platelets

*VIF* variance inflation factor, *SA* spinal anesthesia, *GA* general anesthesia

## Discussion

Postoperative blood loss after TKA is derived from bone and soft tissue, and we could not determine which component generated greater blood loss. Low BMD related to osteoporosis is a common skeletal disorder that is characterized by small bone size and disrupted macro- and micro- architectures [27]. This study demonstrated an association between BMD and the postoperative drainage volume following primary TKA. Participants with a normal total hip BMD had a greater postoperative drainage volume than those with a low total hip BMD. The results of this study showed the linkage of bone quality with postoperative blood loss after primary TKA. The vascular supply in the lower extremities was proposed as a reason for this finding, as a low vascular supply may cause a low total hip BMD and low blood loss during surgery. The linkage between the peripheral vascular supply and BMD has been identified by many previous reports [28–31]. Changes in the hip BMD are associated with blood flow to the lower extremities. Decreased BMD at the hip and calcaneus is associated with decreased vascular flow to the lower extremities in healthy, older women [32]. Thus, this low blood supply may cause a low BMD and reduce blood loss post-TKA. However, to determine the exact pathogenesis of this result, further study is required.

Previous studies reported that large blood sinusoids in trabecular bone are commonly found in patients with osteoporosis and lead to increased bleeding during spinal surgery. Patients with low BMD in the spine were found to have greater perioperative blood loss than those with normal BMD in the spine (357.2 ml vs 259.4 ml) during spinal fusion surgery [20]. On the other hand, lumbar spine BMD status was not found to be associated with the postoperative drainage volume following TKA in this study. The local bone architecture and local soft tissue blood supply may be important factors in blood loss. Hip BMD may be correlated more closely with the bone architecture around the knee joints than the spine, so only hip BMD was associated with blood loss after TKA. Moreover, spinal surgery and TKA use different bleeding-control techniques. In this study, a tourniquet was used to reduce the local soft tissue blood supply and may have played an essential role in controlling bleeding. In addition, the use of a tourniquet and covering of the surface of the bone cut with prosthesis were applied in TKA to decrease blood loss during the operation.

Additionally, this study showed that spinal anesthesia and the operating time were predictive factors associated with an increased postoperative drainage volume in primary TKA. A previous study demonstrated an association between blood loss and type of anesthesia [1]. Similarly, this study supported the finding that regional anesthesia is significantly associated with blood loss in primary TKA. Regarding the operative time, a previous study reported that a longer surgery time is associated with a greater blood transfusion requirement [33]. This study also supported the idea that an increased operative time results in a greater postoperative drainage volume.

This study has several limitations. Firstly, there were missing data, such as blood coagulation results and osteoporosis treatment; also, bias could have existed because of the retrospective nature of the study. Moreover, data on the severity of knee osteoarthritis, degree of soft tissue releases, or peripheral vascular disease were not collected, and this could have been a confounding factor. Secondly, BMD was not directly measured from the distal femur and proximal tibia around the knee joint. However, a previous study reported positive correlations among the distal femur BMD, proximal tibia BMD, and central BMD [34]. The distal femur BMD and proximal tibia BMD should be analyzed to determine their correlations with post-TKA blood loss. Thirdly, the 24-h postoperative drainage volume was included in this analysis. Hidden blood loss may yield a greater volume than visible blood loss [35]. Hemoglobin level may be another useful value to represent total blood loss. However, postoperative hemoglobin level was not routinely measured in our setting, so we did not use this value in the analysis.

## Conclusions

This study demonstrated the association between normal total hip BMD and an increased postoperative drainage volume after primary TKA. Additionally, the study showed that spinal anesthesia and the operating time were predictive factors associated with an increased postoperative drainage volume. The concern about type of anesthesia and shorter operative time should be emphasized in normal total hip BMD patients to reduce the postoperative drainage volume after TKA.

## Data Availability

The datasets used and/or analyzed during the current study are available from the corresponding author on reasonable request.

## Abbreviations

ASA: American Society of Anesthesiologists
BMD: Bone mineral density
BMI: Body mass index
β: Standardized coefficient
DXA: Dual-energy X-ray absorptiometry
GA: General anesthesia
MAP: Mean arterial pressure
*R*^2^: Percent variance explained by each variable
SA: Spinal anesthesia
SD: Standard deviation
SE: Standard error
*t*: Corresponding *t* value
TKA: Total knee arthroplasty
VIF: Variance inflation factor
